# Perioperative outcomes after laparoscopic cholecystectomy in elderly patients: a systematic review and meta-analysis

**DOI:** 10.1007/s00464-020-07805-z

**Published:** 2020-07-13

**Authors:** Sivesh K. Kamarajah, Santhosh Karri, James R. Bundred, Richard P. T. Evans, Aaron Lin, Tania Kew, Chinenye Ekeozor, Susan L. Powell, Pritam Singh, Ewen A. Griffiths

**Affiliations:** 1 Department of Hepatobiliary, Pancreatic and Transplant Surgery, Freeman Hospital, Newcastle University NHS Foundation Trust Hospitals, Newcastle Upon Tyne, UK; 2 Institute of Cellular Medicine, University of Newcastle, Newcastle Upon Tyne, UK; 3 College of Medical and Dental Sciences, University of Birmingham, Birmingham, UK; 4 Department of Upper Gastrointestinal Surgery, Queen Elizabeth Hospital Birmingham, University Hospitals Birmingham NHS Foundation Trust, Area 6, 7th Floor, Mindelsohn Way, Edgbaston, Birmingham, B15 2WB UK; 5 Institute of Cancer and Genomic Sciences, College of Medical and Dental Sciences, University of Birmingham, Birmingham, UK; 6 Department of Geriatric Medicine, Solihull Hospital, University Hospitals Birmingham NHS Foundation Trust, Birmingham, UK; 7 Trent Oesophago-Gastric Unit, City Hospital Campus, Nottingham University Hospitals NHS Trust, Hucknall Road, Nottingham, NG5 1PB UK; 8 Regional Oesophago-Gastric Unit, Royal Surrey County Hospital NHS Foundation Trust, Egerton Road, Guildford, GU2 7XX UK

**Keywords:** Cholecystectomy, Elderly, Outcomes, Laparoscopic

## Abstract

**Background:**

Laparoscopic cholecystectomy is increasingly performed in an ever ageing population; however, the risks are poorly quantified. The study aims to review the current evidence to quantify further the postoperative risk of cholecystectomy in the elderly population compared to younger patients.

**Method:**

A systematic literature search of PubMed, EMBASE and the Cochrane Library databases were conducted including studies reporting laparoscopic cholecystectomy in the elderly population. A meta-analysis was reported in accordance with the recommendations of the Cochrane Library and PRISMA guidelines. Primary outcome was overall complications and secondary outcomes were conversion to open surgery, bile leaks, postoperative mortality and length of stay.

**Results:**

This review identified 99 studies incorporating 326,517 patients. Increasing age was significantly associated with increased rates of overall complications (OR 2.37, CI_95%_ 2.00–2.78), major complication (OR 1.79, CI_95%_ 1.45–2.20), risk of conversion to open cholecystectomy (OR 2.17, CI_95%_ 1.84–2.55), risk of bile leaks (OR 1.50, CI_95%_ 1.07–2.10), risk of postoperative mortality (OR 7.20, CI_95%_ 4.41–11.73) and was significantly associated with increased length of stay (MD 2.21 days, CI_95%_ 1.24–3.18).

**Conclusion:**

Postoperative outcomes such as overall and major complications appear to be significantly higher in all age cut-offs in this meta-analysis. This study demonstrated there is a sevenfold increase in perioperative mortality which increases by tenfold in patients > 80 years old. This study appears to confirm preconceived suspicions of higher risks in elderly patients undergoing cholecystectomy and may aid treatment planning and informed consent.

**Electronic supplementary material:**

The online version of this article (10.1007/s00464-020-07805-z) contains supplementary material, which is available to authorised users.

Over 66,000 cholecystectomies are performed each year in the UK costing over £110 million to the National Health Service [1, 2]. The majority of these cases are now done laparoscopically, owing to significantly lower rates of morbidity and mortality compared to conventional open surgery. Recently, a national multicentre study highlighted that 96% of cases are done laparoscopically, establishing laparoscopic cholecystectomy as mainstay management for various benign gallbladder diseases [3, 4]. Biliary colic or acute cholecystitis accounts for > 70% of the indications for performing a cholecystectomy.

With an ageing population, demands for surgery are expected to rise over the next decade with associated increasing frailty [5, 6]. In parallel, it is estimated that increasing numbers of elderly patients will present with gallstone disease [7, 8], and it is thought that patients aged 80–89 years account for 28% and 42% of male and female patients, respectively [8, 9]. Since age is associated with existence of multiple comorbidities and reduced functional reserve, it is thought that operating on elderly patients could be associated with an increased risk of complications [10–12]. Despite this, elderly patients may still undergo laparoscopic cholecystectomy. Many studies evaluating outcomes of laparoscopic cholecystectomy in their definition of elderly patients versus younger patients have shown that increasing age leads to higher conversion rates, more complications and a longer hospital stay [7–10].

Despite this, current literature on definition of an elderly population in regard to outcomes following laparoscopic cholecystectomy is heterogeneous. Hence, counselling elderly patients regarding morbidity and mortality of laparoscopic cholecystectomy is difficult. To date, current evidence is limited to cohort studies and no meta-analysis characterising the impact of age on postoperative complications following laparoscopic cholecystectomy exists. Therefore, this study aimed to perform a systematic review and meta-analysis to summarise postoperative outcomes of elderly patients undergoing laparoscopic cholecystectomy.

## Methods

This paper is reported according to the PRISMA guidelines [13] and was prospectively registered with the PROSPERO database (Registration CRD42019125343). This study did not require approval by the Institutional Review Board as this was a systematic review of current literature and no patient consent was required. There was deviation from the protocol with additional analyses by urgency of surgery and the use of ROBINS-I for study quality assessment.

### Search strategy

A systematic search of PubMed, EMBASE and the Cochrane Library databases was conducted on the 1 November 2018 by two independent investigators (SKK, SK). The search terms used were ‘cholecystectomy’ or ‘laparoscopic’, and ‘treatment outcome’ or ‘complications’ or ‘intraoperative complications’ or ‘postoperative complication’, and ‘elderly’ or ‘octogenarian’ or ‘frail’ individually or in combination. Search terms used for this review are presented as shown in Supplementary Table 1. The ‘related articles’ function was used to broaden the search, and all citations were considered for relevance. A manual search of reference lists in recent reviews and eligible studies was also undertaken.

### Inclusion and exclusion criteria

The inclusion criteria were (1) studies reporting comparative analysis of laparoscopic cholecystectomy in elderly patients; (2) published in the English language. Studies were still included even if all the relevant outcome data were not provided. Exclusion criteria were (1) conference abstracts, systematic reviews, meta-analyses and case reports (< 5 patients); (2) any studies that did not report the outcomes of the surgery numerically. Two independent authors (RSK, SK) excluded duplicates and independently reviewed the titles, abstracts and full articles of the studies identified by the literature search. Where the full article was not available, in paper or electronic form, the article was excluded. Major reviews were manually searched to obtain any other potentially relevant studies. The authors discussed any cases which presented any differences.

### Study outcomes

The primary outcome measures were overall complications (Grade I–V) and major complications (≥ Grade III) reported according to Clavien–Dindo Classification [14]. Secondary outcomes were conversion to open surgery, bile leaks, perioperative mortality and length of hospital stay.

### Data extraction

Three authors extracted data independently and reviewed the data for any discrepancies. The data collected included study characteristics (authors, year of publication, country of origin, patient number, definition of elderly, type of study), indications for surgery and outcome measures (overall and major complications, major complications, conversion, bile leaks, perioperative mortality, length of hospital stay).

### Assessment of methodological quality

Four independent authors (SKK, RSK, CE, TC) assessed the methodological quality using the risk of bias in non-randomised studies of interventions (ROBINS-I) [15]. The ROBINS-I assessed each study on seven distinct domains through which bias might be introduced. These domains are divided according to pre-intervention (bias due to confounding and bias in selection of participants into the study), at intervention (bias in classification of interventions), and post-intervention (bias due to deviations from intended interventions, bias due to missing data, bias in measurement of outcomes and bias in selection of the reported result). Each of these seven domains is graded according to low, moderate, critical, serious or no information.

### Statistical methods

This systematic review and meta-analysis was conducted in accordance with the recommendations of the Cochrane Library and PRISMA guidelines [13]. For binary variables, analysis was performed by calculating the odds ratio (OR). For continuous data, analysis was performed to calculate the cumulative mean difference (MD) with 95% confidence intervals (CI_95%_). The random effects, the DerSimonian–Laird method, was used for the meta-analysis of outcomes. Heterogeneity between studies was assessed using the *I*_2_ value in order to determine the degree of variation not attributable to chance alone. *I*_2_ values were considered to represent low, moderate and high degrees of heterogeneity where values were < 25%, 25–75% and > 75%, respectively. Assessment of small study effects was carried out by visual assessment of funnel plots and Egger regressions. Stratified analyses were performed by urgency of surgery (i.e. elective and emergency) for each postoperative outcome. Statistical significance was considered when *p* < 0.05. Statistical analyses were performed using the RevMan 5.3 software (Copenhagen: The Nordic Cochrane Centre, The Cochrane Collaboration, 2011) and R statistical software (Version 3.5.2, R Foundation for Statistical Computing, Vienna, Austria) as previously described [16, 17].

## Results

### Study characteristics

**Fig. 1:**
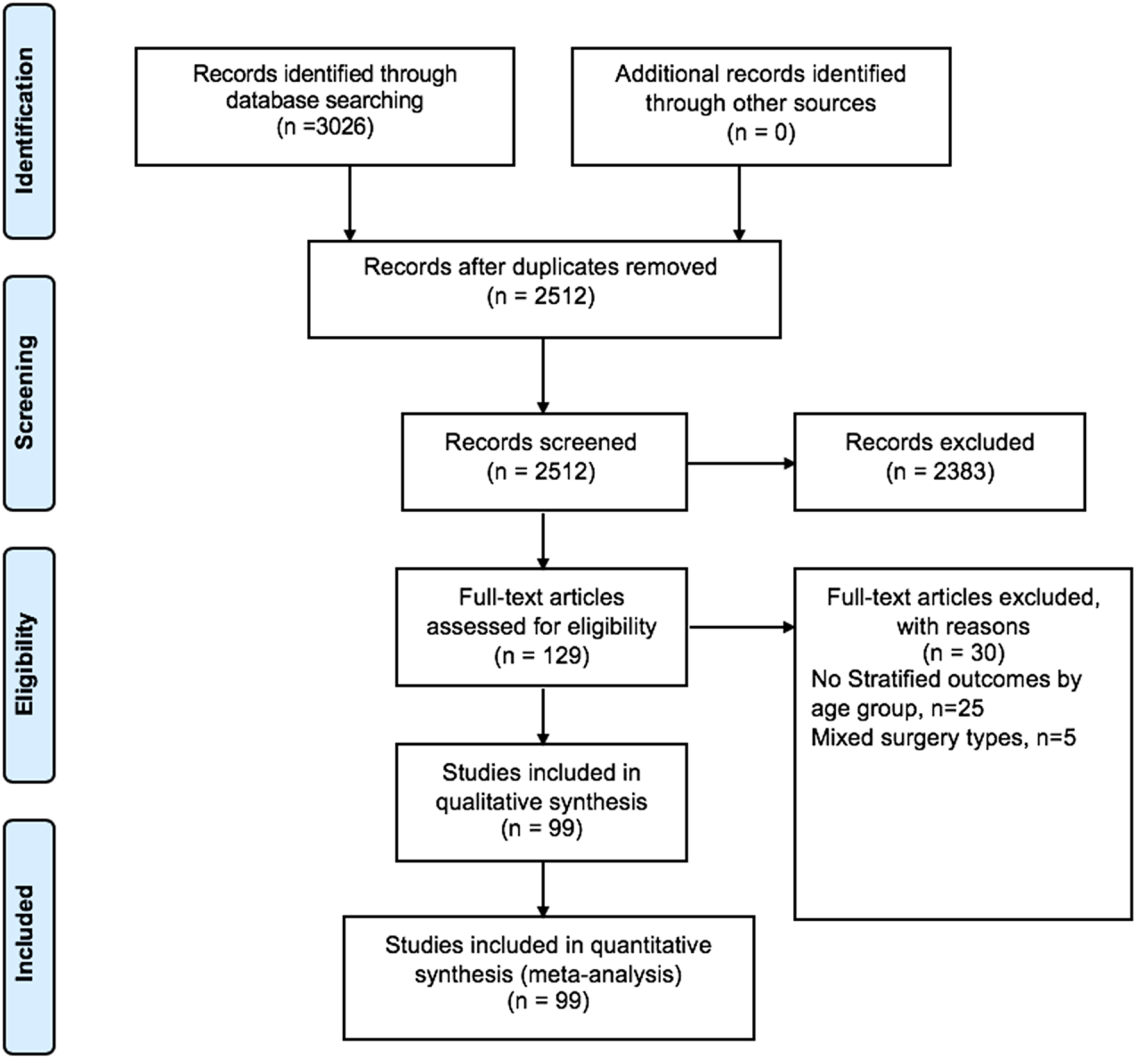
PRISMA diagram of included studies

This study included 99 studies (*n* = 326,517 patients) reported according to the PRISMA guidelines as shown in Fig. 1. Baseline characteristics of studies are presented in Supplementary Table 2. Studies identified were from North America (*n* = 28), Asia (*n* = 31) and Europe (*n* = 39). Indication for surgery was reported only in 77 studies. Urgency of cholecystectomy were reported in 90 studies (*n* = 303,463 patients), of which 7% (*n* = 19,754) were elective cases and 67% (*n* = 203,924) were emergency cases. In this study, 52 studies were comparative studies.

### Reporting standards and methodological quality

Study quality was assessed using ROBINS-I for comparative studies, with majority of studies (78%, *n* = 77/99) deemed as overall low risk of bias (Supplementary Table 3). There was significant variation in cut-off ages used to define elderly populations with age ≥ 60 (*n* = 8 studies), ≥ 65 (*n* = 39 studies), ≥ 70 (*n* = 14 studies), ≥ 75 (*n* = 11 studies), ≥ 80 (*n* = 22 studies) the most commonly used. Studies were pooled into subgroups according to age cut-off value.

### Overall complications

**Fig. 2:**
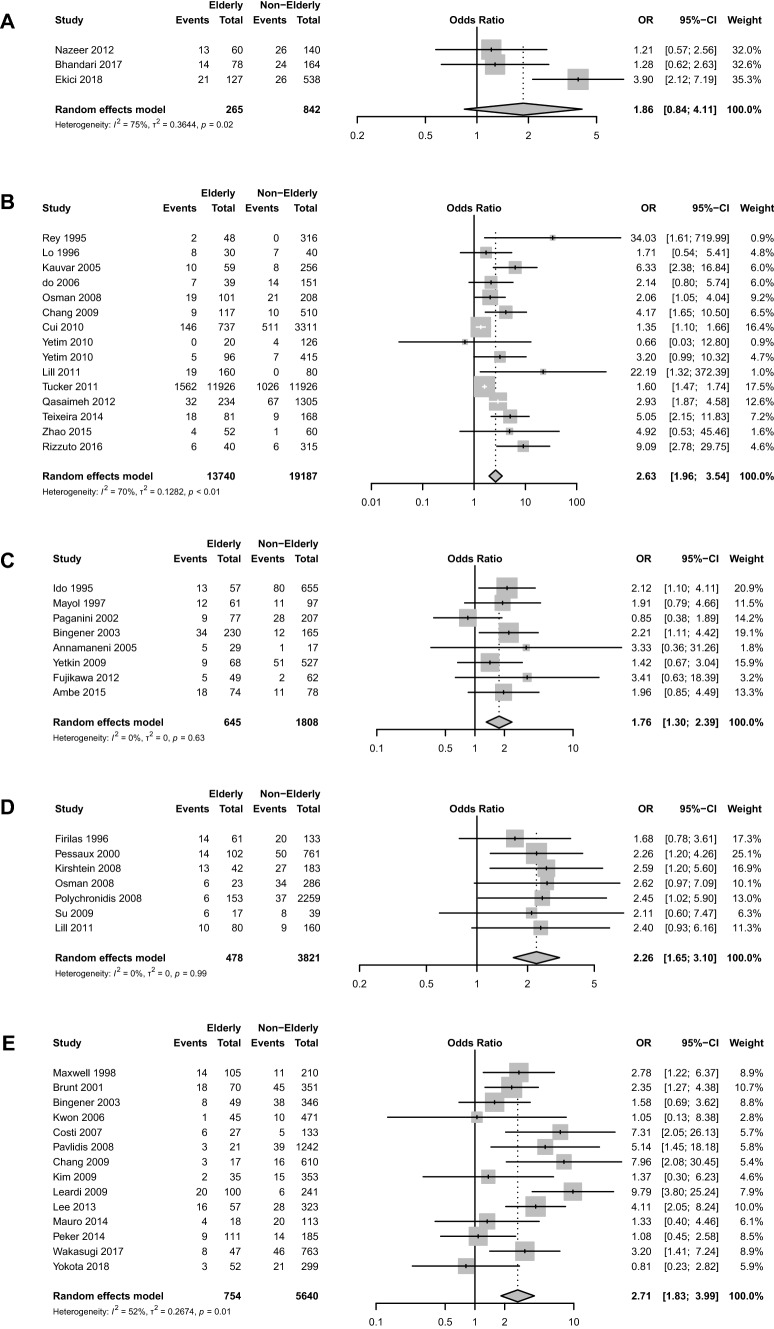
Impact of age cut-offs on overall complications in patients undergoing laparoscopic cholecystectomy **A** ≥ 60 years **B** ≥ 65 years **C** ≥ 70 years **D** ≥ 75 years **E** ≥ 80 years

Impact of increasing age on overall complications was reported in 48 studies, including 49,215 patients. Overall, increasing age was significantly associated with increased rates of overall complications (OR 2.37, CI_95%_ 2.01–2.78, *I*_2_ = 56%) (Supplementary Fig. 1). Overall complication rates were significantly increased in age subgroups: ≥ 65 years (OR 2.63, CI_95%_ 1.96–3.54, *I*_2_ = 70%), ≥ 70 years (OR 1.76 CI_95%_ 1.30–2.39, *I*_2_ = 0%), ≥ 75 years (OR 2.26, CI_95%_ 1.65–3.10, *I*_2_ = 0%) and ≥ 80 (OR 2.71, CI_95%_ 1.83–3.99, *I*_2_ = 52%) (Table 1, Fig. 2). When stratified by urgency, increasing age was associated with significantly higher overall complications for elective (OR 2.46, CI_95%_ 1.63–3.71, *I*_2_ = 38%) and emergency (OR 1.98, CI_95%_ 1.33–2.94, *I*_2_ = 48%) laparoscopic cholecystectomy (Table 2).

**Table 1:** Summary of postoperative outcomes in patients undergoing laparoscopic cholecystectomy

Outcomes	Studies, *n*	Patients, *n*	Odds ratio/mean difference* (95% CI)	*p* value	*I*^2^
Overall complications					
≥ 60 vs < 60 years	3	1107	1.86 (0.84–4.11)	0.1	75
≥ 65 vs < 65 years	15	32,927	2.63 (1.96–3.54)	< 0.001	70
≥ 70 vs < 70 years	8	2453	1.76 (1.30–2.39)	< 0.001	0
≥ 75 vs < 75 years	7	4299	2.26 (1.65–3.10)	< 0.001	0
≥ 80 vs < 80 years	14	6394	2.71 (1.83–3.99)	< 0.001	52
Major complications					
≥ 60 vs < 60 years	0		–	–	–
≥ 65 vs < 65 years	2	15,044	1.51 (1.38–1.65)	< 0.001	0
≥ 70 vs < 70 years	3	831	2.16 (0.71–6.53)	0.2	71
≥ 75 vs < 75 years	2	919	1.88 (0.35–10.14)	0.5	0
≥ 80 vs < 80 years	7	17,954	1.95 (1.65–2.29)	< 0.001	0
Conversion to Open					
≥ 60 vs < 60 years	3	1107	1.52 (0.91–2.50)	0.1	0
≥ 65 vs < 65 years	18	27,122	2.62 (1.97–3.49)	< 0.001	63
≥ 70 vs < 70 years	6	1695	1.50 (0.54–4.13)	0.4	78
≥ 75 vs < 75 years	7	4519	2.04 (1.54–2.69)	< 0.001	0
≥ 80 vs < 80 years	17	22,340	2.48 (1.97–3.12)	< 0.001	13
Bile Leaks					
≥ 60 vs < 60 years	2	442	0.56 (0.12–2.69)	0.5	0
≥ 65 vs < 65 years	10	20,889	1.43 (0.93–2.23)	0.1	0
≥ 70 vs < 70 years	4	1189	1.60 (0.62–4.13)	0.3	0
≥ 75 vs < 75 years	5	3127	1.78 (0.49–6.41)	0.4	0
≥ 80 vs < 80 years	9	17,118	2.05 (0.87–4.84)	0.1	0
Postoperative mortality					
≥ 60 vs < 60 years	2	442	–	–	–
≥ 65 vs < 65 years	16	49,962	5.17 (2.48–10.77)	< 0.001	56
≥ 70 vs < 70 years	6	1583	4.86 (1.04–22.75)	0.045	0
≥ 75 vs < 75 years	9	4795	6.09 (1.61–23.02)	0.008	2
≥ 80 vs < 80 years	15	21,185	10.20 (4.97–20.92)	< 0.001	32
Length of Stay, days					
≥ 60 vs < 60 years	–		–	–	–
≥ 65 vs < 65 years	7	2920	2.33 (1.10–3.56)	< 0.001	96
≥ 70 vs < 70 years	5	1616	3.37 (0.28–6.46)	0.032	98
≥ 75 vs < 75 years	4	2933	1.08 (− 0.28–2.44)	0.1	92
≥ 80 vs < 80 years	7	3173	1.33 (0.54–2.11)	< 0.001	83

*Mean difference were used for the presentation of length of stay data

**Table 2:** Summary postoperative outcomes in elderly and non-elderly patients by urgency of laparoscopic cholecystectomy

Outcomes	Studies, *n*	Patients, *n*	Odds ratio (95% CI)	*p* value	*I*^2^
Overall complications					
Elective	10	4630	2.46 (1.63–3.71)	< 0.001	38
Emergency	8	5396	1.98 (1.33–2.94)	< 0.001	48
Mixed	24	36,324	2.27 (1.83–2.82)	< 0.001	52
Major complications					
Elective	1	284	–	–	–
Emergency	1	152	–	–	–
Mixed	10	33,058	1.81 (1.47–2.21)	< 0.001	35
Conversion to open					
Elective	9	4630	2.48 (1.71–3.59)	< 0.001	0
Emergency	11	6414	2.28 (1.39–3.75)	0.001	58
Mixed	27	44,069	2.09 (1.67–2.63)	< 0.001	69
Bile leaks					
Elective	4	442	0.56 (0.12–2.69)	0.5	0
Emergency	8	5819	1.28 (0.75–2.18)	0.3	0
Mixed	15	34,603	1.53 (0.95–2.45)	0.1	0
Postoperative mortality					
Elective	9	2595	13.34 (2.07–85.92)	0.006	0
Emergency	11	6414	5.54 (1.96–15.70)	0.001	0
Mixed	25	65,482	8.08 (4.37–14.92)	< 0.001	73
Length of stay, days					
Elective	4	752	1.84 (1.27–2.41)	< 0.001	64
Emergency	7	1513	2.63 (1.13–4.14)	< 0.001	88
Mixed	5	8020	1.99 (0.44–3.54)	0.012	99

### Major complications

Impact of increasing age on major complications was reported in 14 studies, including 34,748 patients. Increasing age was associated with significantly higher rates of major postoperative complications (OR 1.79, CI_95%_ 1.45–2.20, *I*_2_ = 34%) (Supplementary Fig. 2). Major complication rates were significantly increased in subgroups: ≥ 65 years (OR 1.51, CI_95%_ 1.38–1.65, *I*_2_ = 0%) and ≥ 80 years (OR 1.95, CI_95%_ 1.65–2.29, *I*_2_ = 0%) (Supplementary Fig. 3). Since there was only one study reporting outcomes for major complications elective and emergency laparoscopic cholecystectomy, subgroup analyses were not possible (Table 2).

### Conversion to open surgery

Impact of increasing age on conversion to open cholecystectomy was reported in 53 studies, including 59,173 patients. Overall, increasing age was significantly associated with increased odds of conversion to open cholecystectomy (OR 2.17, CI_95%_ 1.84–2.55, *I*_2_ = 56%) (Supplementary Fig. 4). Conversion to open cholecystectomy was significantly increased in subgroups with the following age cut-offs: ≥ 65 (OR 2.62, CI_95%_ 1.97–3.49, *I*_2_ = 63%), ≥ 75 (OR 2.04, CI_95%_ 1.54–2.69, *I*_2_ = 0%), ≥ 80 (OR 2.48, CI_95%_ 1.97–3.12, *I*_2_ = 13%) (Table 1, Supplementary Fig. 5). When stratified by urgency, increasing age was associated with significantly higher odds for conversion for elective (OR 2.48, CI_95%_ 1.71–3.59, *I*_2_ = 0%) and emergency (OR 2.28, CI_95%_ 1.39–3.75, *I*_2_ = 58%) laparoscopic cholecystectomy (Table 2).

### Bile leaks

Impact of increasing age on bile leaks was reported in 30 studies, including 42,765 patients. Overall, increasing age was significantly associated with increased rates of bile leaks (OR 1.50, CI_95%_ 1.07–2.10, *I*_2_ = 0%) (Supplementary Fig. 6). However, bile leaks were not increased in studies reporting age in all age cut-offs (Table 1, Supplementary Fig. 7). When stratified by urgency, increasing age was not associated with higher rates of bile leaks for elective (OR 0.56, CI_95%_ 0.12–2.69, *I*_2_ = 0%) and emergency (OR 1.28, CI_95%_ 0.75–2.18, *I*_2_ = 0%) laparoscopic cholecystectomy (Table 2).

### Postoperative mortality

**Fig. 3:**
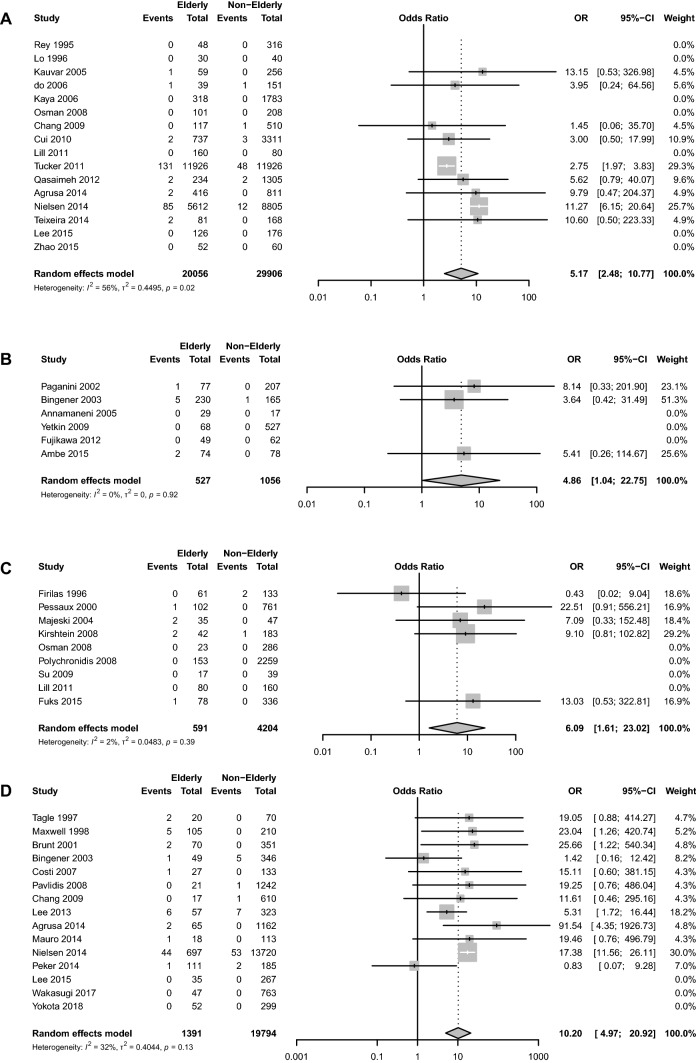
Impact of age cut-offs on bile leaks in patients undergoing laparoscopic cholecystectomy **A** ≥ 65 years **B** ≥ 70 years **C** ≥ 75 years **D** ≥ 80 years

Impact of increasing age on postoperative mortality was reported in 50 studies, including 78,404 patients. Overall, increasing age was significantly associated with increased rates of postoperative mortality (OR 7.20, CI_95%_ 4.41–11.73, *I*_2_ = 59%) (Supplementary Fig. 8). Postoperative mortality rates were significantly increased in all age cut-off subgroups: ≥ 65 (OR 5.17, CI_95%_ 2.48–10.77, *I*_2_ = 56%), ≥ 70 (OR 4.86, CI_95%_ 1.04–22.75, *I*_2_ = 0%), ≥ 75 (OR 6.09, CI_95%_ 1.61–23.02, *I*_2_ = 2%), ≥ 80 (OR 10.20, CI_95%_ 4.97–20.92, *I*_2_ = 32%) (Fig. 3). When stratified by urgency, increasing age was associated with significantly higher rates of postoperative mortality for elective (OR 13.34, CI_95%_ 2.07–85.92, *I*_2_ = 0%) and emergency (OR 5.54, CI_95%_ 1.96–15.70, *I*_2_ = 0%) laparoscopic cholecystectomy (Table 2).

### Length of stay

Impact of increasing age on length of stay following cholecystectomy was reported in 24 studies, including 10,997 patients. Overall, increasing age was significantly associated with increased length of stay (Mean difference (MD): 2.21, CI_95%_ 1.24–3.18, *I*_2_ = 99%) (Supplementary Fig. 9). Length of stay was significantly increased in age cut-off subgroups: ≥ 65 (MD 2.33, CI_95%_ 1.10–3.56, *I*_2_ = 96%), ≥ 70 (MD 3.37, CI_95%_ 0.28–6.46, *I*_2_ = 98%) and ≥ 80 (MD 1.33, CI_95%_ 0.54–2.11, *I*_2_ = 83%) (Table 1, Supplementary Fig. 10). When stratified by urgency, increasing age was associated with significantly longer length of stay for elective (MD 1.84, CI_95%_ 1.27–2.41, *I*_2_ = 64%) and emergency (MD 2.63, CI_95%_ 1.13–4.14, *I*_2_ = 88%) laparoscopic cholecystectomy (Table 2).

### Publication biases

Egger regression testing suggested publication biases were minimal for reporting of major complications (*p* = 0.314, Supplementary Fig. 11), conversion to open (*p* = 0.352, Supplementary Fig. 12), bile leaks (*p* = 0.589, (Supplementary Fig. 13) and postoperative mortality (*p* = 0.172, (Supplementary Fig. 14), and length of stay (*p* = 0.728, Supplementary Fig. 15). Egger regression testing for publication bias was significant for reporting of overall complications (*p* = 0.004, Supplementary Fig. 16). Subgroup analyses showed that publication biases were present in the subgroup of studies reporting age > 65 as a cut-off (*p* = 0.0012) and not in any of the other subgroups.

#### Sensitivity analysis of adjusted outcomes

Heterogeneity in the effect of increasing age on the reported outcomes was hypothesised to be due to the wide-range of age cut-offs used. Subgroup analyses of studies reporting only age cut-offs ≥ 70 and ≥ 75 were therefore completed.

## Discussion

This systematic review and meta-analysis including 99 studies and 326,517 patients undergoing laparoscopic cholecystectomy demonstrated postoperative outcomes such as overall and major complications (Clavien–Dindo ≥ Grade III) were significantly higher in all age cut-offs. Further, this study demonstrated there is a sevenfold increase in perioperative mortality in the elderly compared to the non-elderly which increases by tenfold in patients ≥ 80 years old. Although the risk of bile leak was higher in the overall cohort, there were no significant differences in bile leak in age cut-offs of ≥ 70 and ≥ 80 years old, respectively. There were significantly higher rates of conversions, as high as threefold in the elderly population. Patients converted from a minimally access surgery to open surgery have a higher risk of perioperative mortality and morbidity, as demonstrated in this review [18–20]. These findings were consistent even when stratified by urgency of surgery (i.e. elective or emergency). Whilst current risk estimation considers presence of comorbidities or American Society of Anaesthesiologist (ASA) grade, adopting risk profiles of varying age groups should also be considered during pre-operative counselling. Pre-operative optimisation of comorbidities, medications, screening for dementia and addressing frailty by a geriatrician or a full multi-disciplinary team could help improve operative outcomes in these patients [21–23].

The proportion of the population over the age of 65 is projected to rise from 18% currently to 25% in 30 years’ time. Gallstone disease more commonly affects the elderly and in turn the sequelae of gallstone disease such as biliary colic, cholecystitis and pancreatitis make up a large aspect of acute surgical admissions. Current guidance advocates acute cholecystectomy within 1 week from the onset of acute cholecystitis and within 2 weeks if gallstones are the precipitant cause of pancreatitis [24]. Cholecystectomy in the acute setting has been shown to be both safe and cost effective [4, 25]. Increasing evidence also suggests superiority of cholecystectomy over cholecystostomy in high-risk patients [26, 27]. Patients undergoing cholecystostomy are predominantly elderly and up to a quarter of patients undergoing cholecystostomy will be re-admitted within 30 days of discharge [28]. Elderly patients undergoing cholecystostomy have been shown to have higher rates of mortality, post-procedural infection, bleeding and length of stay as compared to cholecystectomy [29]. These factors are changing the way we interpret and treat gallstone disease leading to a rise in the number of cholecystectomies performed each year. This highlights the importance in understanding and defining the impact of laparoscopic cholecystectomy on the elderly population.

This systematic review and meta-analysis adds evidence to quantify risk in elderly populations undergoing cholecystectomy. Increasingly healthcare professionals are questioned about discriminating against patients due to their age. It is often assumed that age is synonymous with increased co-morbidity. Due to lack of reporting in numerous studies, it was not possible to analyse age independently of co-morbidity. Future studies are increasingly adjusting for comorbidities and this is a potential area for enhancement in subsequent reviews. The meta-analysis is challenged by the lack of reporting of randomised and multicentre publications which is a further source of potential improvement with the publication of higher quality evidence. Despite some of the potential areas for improvement, this meta-analysis is the first to specifically quantify the postoperative risk of cholecystectomy in the elderly population. It encompasses a large number of studies which in turn incorporates over 300,000 patients. As the population ages, it is important that we improve our understanding of risk for elderly patients undergoing cholecystectomy and this meta-analysis makes significant strides to achieve this.

Consideration of alternative treatment strategies to laparoscopic in elderly patients will aid in informed-decision making amongst patients. In patients with acute cholecystitis, alternative strategies may include antibiotic management and percutaneous catheter drainage [30, 31]. A recent randomised controlled trial [26] comparing percutaneous catheter drainage and laparoscopic cholecystectomy for high-risk patients with acute cholecystitis identified laparoscopic cholecystectomy to be associated with significantly lower rates of major complications and re-interventions, although no significant difference in mortality rates. Whilst bridging with percutaneous catheter drainage prior to laparoscopic cholecystectomy may be a sensible treatment strategy, this needs to be evaluated in a case-by-case basis. However, alternative strategies for biliary colic and chronic cholecystitis are conservative options such as diet and weight control to reduce episodes of gallstone related complications. Nonetheless, careful discussion with patients on risks and benefits of laparoscopic cholecystectomy is warranted.

Our systematic review and meta-analysis do have some limitations to be addressed. Firstly, whilst it is a thorough review containing large patient numbers, the evidence comes from mainly retrospective case series and from studies which have used different age cut-offs. Secondly, chronological age has many limitations and data on biological fitness is lacking to explain why elderly patients develop poorer outcomes compared to younger patients [32]. For example, prospective scoring of patient frailty, an increasingly recognised feature associated with poor postoperative outcomes, is lacking [7, 33]. Thirdly, a precise scoring of intraoperative cholecystectomy difficulty is lacking [34, 35]. Elderly patients often have gallstones for many years and features of chronic cholecystitis with the obliteration of the tissue planes in Calot’s triangle and even chronic fistulation, such as Mirizzi syndrome [36] or cholecysto-enteric fistulation, which naturally lead to poorer patient outcomes [37]. These challenging operative conditions require a modification in surgical strategies to ensure best patient outcomes [38]. Finally, whilst length of stay should ideally come with discharge to a nursing facility, it remains unclear from the included studies if this were the case and hence could not be evaluated.

## Conclusion

Overall and major complications are significantly increased in the elderly following laparoscopic cholecystectomy. There is an associated sevenfold increase in perioperative mortality which increases to tenfold in patients ≥ 80 years old group. This study confirms preconceived suspicions of higher risks in elderly patients undergoing cholecystectomy and will aid treatment planning and informed consent.

## Electronic supplementary material

Below is the link to the electronic supplementary material.

Supplementary file 1 (DOCX 2114 kb)
