# Supplementary material for: Perioperative outcomes after laparoscopic cholecystectomy in elderly patients: a systematic review and meta-analysis
Source: Surg Endosc. 2020 Jul 13;34(11):4727–40. doi: 10.1007/s00464-020-07805-z (PMC7572343; doi:10.1007/s00464-020-07805-z)
Supplement: Supplementary file 1 — (DOCX 2114 kb) [file 464_2020_7805_MOESM1_ESM.docx]

**Perioperative Outcomes after Laparoscopic Cholecystectomy in Elderly Patients: A Systematic Review and Meta-Analysis**

Sivesh K Kamarajah^1, 2*^, Santhosh Karri^3*^, James R Bundred^3#^, Richard PT Evans^4, 5#,^ Aaron Lin^3^, Tania Kew^3^, Chinenye Ekeozor^3^, Susan L Powell ^6^, Pritam Singh^7,8^, Ewen A Griffiths^4, 5^

1. Department of Hepatobiliary, Pancreatic and Transplant Surgery, Freeman Hospital, Newcastle University NHS Foundation Trust Hospitals, Newcastle Upon Tyne, UK
2. Institute of Cellular Medicine, University of Newcastle, Newcastle Upon Tyne, UK
3. College of Medical and Dental Sciences, University of Birmingham, Birmingham, UK
4. Department of Upper Gastrointestinal Surgery, University Hospitals Birmingham NHS Foundation Trust, Birmingham, UK
5. Institute of Cancer and Genomic Sciences, College of Medical and Dental Sciences, University of Birmingham, UK
6. Department of Geriatric Medicine, Solihull **Hospital**, University Hospitals Birmingham NHS Foundation Trust, Birmingham, UK
7. Trent Oesophago-Gastric Unit, City Hospital Campus, Nottingham University Hospitals NHS Trust, Hucknall Road, Nottingham NG5 1PB, U.K.
8. **Regional Oesophago-Gastric Unit, Royal Surrey County Hospital NHS Foundation Trust, Egerton Road, Guildford, GU2 7XX**

* these authors contribute equally as co-first authors

# these authors contribute equally as co-second authors

**Corresponding Author Contact Details:**

Mr. Ewen A Griffiths,

Consultant Upper GI Surgeon,

Department of Upper Gastrointestinal Surgery,

Area 6, 7th Floor, Queen Elizabeth Hospital Birmingham,

Mindelsohn Way, Edgbaston, Birmingham

B15 2WB

Email: [ewen.griffiths@uhb.nhs.uk](mailto:ewen.griffiths@uhb.nhs.uk)

Telephone: 0121 3715886

Fax: 0121 371 5896

**Number of pages: 14**

**Word Count: 2931**

**Number of tables: 2**

**Number of figures: 3**

**Keywords:** cholecystectomy; elderly; outcomes; laparoscopic

Supplementary Table 1 Search terms in literature review

| 1 | cholecystectomy.ti,ab. | 67414 |
| --- | --- | --- |
| 2 | exp Treatment Outcome/ | 2360254 |
| 3 | complications.ti,ab. | 1765338 |
| 4 | exp INTRAOPERATIVE COMPLICATIONS/ or exp POSTOPERATIVE COMPLICATIONS/ | 1143356 |
| 5 | 2 or 3 or 4 | 4551783 |
| 6 | exp "aged, 80 and over"/ or exp frail elderly/ | 973162 |
| 7 | elderly.ti,ab. | 684607 |
| 8 | octogenarian.ti,ab. | 3375 |
| 9 | 6 or 7 or 8 | 1563007 |
| 10 | 1 and 5 and 9 | 3026 |

Supplementary Table 2 Baseline characteristics of included studies

| **Study Name** | **Region** | **Patients, n** | **Study Design** | **Indications** | | | | | | **Urgency** | | **Elderly Definition, years** | **Comparative Study** |
| --- | --- | --- | --- | --- | --- | --- | --- | --- | --- | --- | --- | --- | --- |
|  |  |  |  | **Biliary colic, n** | **Acute cholecystitis, n** | **Chronic cholecystitis, n** | **Gallstones, n** | **Gallstone pancreatitis, n** | **Others, n** | **Emergency** | **Elective** |  |  |
| Schreiber 1978^39^ | USA | 39 | RCS | - | - | - | 39 | - | - | 39 |  | >60 | No |
| Loureiro 2011^40^ | Brazil | 960 | RCS | - | 105 | 832 | - | - | 23 | - | - | >60 | No |
| Serban 2016^36^ | Romania | 149 | RCS | - | 149 | - | - | - | - | - | - | >60 | No |
| Houghton 1985^41^ | UK | 151 | PCS | - | - | - | - | - | - | 21 | 130 | >64 | No |
| Amato 2010^42^ | Italy | 121 | RCS | - | 66 | - | 24 | 9 | 44 | - | - | >64 | No |
| Pigott 1988^43^ | USA | 347 | RCS | - | - | - | - | - | - | - | - | >65 | No |
| Saxe 1993^44^ | USA | 94 | RCS | - | - | - | - | - | - | - | - | >65 | No |
| Askew 1995^45^ | Australia | 51 | RCS | - | - | - | 51 | - | - | - | - | >65 | No |
| Escarce 1995^46^ | USA | 21131 | RCS | - | 7121 | 10692 | 63 | 1606 | 1649 | 13418 | 7713 | >65 | No |
| Lujan 1998^47^ | Spain | 264 | RCS | - | - | - | 264 | - | - | - | - | >65 | No |
| Wang 2006^48^ | Taiwan | 112 | RCS | - | 112 | - | - | - | - | - | - | >65 | No |
| Malik 2007^49^ | Pakistan | 173 | RCS | - | - | - | 173 | - | - | - | - | >65 | No |
| Cheng 2008^50^ | Taiwan | 287 | RCS | - | - | - | - | 15 | - | 19 | 268 | >65 | No |
| Lasithiotakis 2012^51^ | Greece | 57 | **PCS** | - | - | 36 | 42 | - | 9 | 0 | 57 | >65 | No |
| Cull 2013^52^ | USA | 265 | RCS | - | 264 | - | - | - | - | - | - | >65 | No |
| Ferrarese 2013^53^ | Italy | 85 | RCS | - | - | - | - | - | - | 40 | 45 | >65 | No |
| Rao 2013^54^ | USA | 15248 | RCS | - | - | - | - | - | - | - | - | >65 | No |
| Rubert 2016^55^ | Brazil | 113 | RCS | - | - | - | - | - | - | - | - | >65 | No |
| Zeren 2017^56^ | Turkey | 45 | RCS | - | - | - | 45 | - | - | - | - | >65 | No |
| Schlottmann 2018^29^ | USA | 193, 339 | RCS | - | 193, 339 | - | - | - | - | - | - | >65 | No |
| Huber 1983^57^ | USA | 93 | RCS | - | - | - | - | - | 93 | 43 | 50 | >70 | No |
| Margiotta 1988^58^ | USA | 212 | RCS | - | 106 | 106 | - | - | - | 93 | 119 | >70 | No |
| Watemberg 1997^59^ | Israel | 68 | RCS | - | - | - | - | - | - | 8 | 60 | >70 | No |
| Montori 2000^60^ | Italy | 31 | RCS | - | - | - | - | - | - | 11 | 20 | >70 | No |
| Fisichella 2002^61^ | Italy | 24 | RCS | - | - | - | - | - | 24 | 0 | 24 | >70 | No |
| Zappulla 2009^62^ | Italy | 40 | RCS | 26 | 9 | - | - | - | 5 | - | - | >70 | No |
| Caglia 2012^63^ | Italy | 50 | RCS | 36 | 10 | - | 50 | 4 | - | - | - | >70 | No |
| Aprea 2016^64^ | Italy | 79 | RCS | - | - | - | - | - | - | - | - | >70 | No |
| Mastalerz 2018^65^ | Poland | 114 | PCS | - | - | - | 105 | - | 37 | 0 | 114 | >70 | No |
| Decker 2001^66^ | Belgium | 27 | RCS | - | 32 | - | - | - | - | 27 | 0 | >75 | No |
| Pessaux 2001^67^ | France | 139 | PCS | - | 139 | - | - | - | - | - | - | >75 | No |
| Chau 2002^68^ | Hong Kong | 296 | RCS | - | 296 | - | - | - | - | - | - | >75 | No |
| Coenye 2005^69^ | Belgium | 20 | RCS | - | 20 | - | - | - | - | - | - | >75 | No |
| Moyson 2008^70^ | Belgium | 100 | RCS | - | 100 | - | - | - | - | 85 | 15 | >75 | No |
| Maxwell 1998^71^ | USA | 18500 | RCS | - | - | - | - | - | - | - | - | >80 | No |
| Uecker 2001^72^ | USA | 44 | RCS | - | 27 | 8 | 22 | 12 | 1 | 28 | 16 | >80 | No |
| Arthur 2003^9^ | UK | 23 | RCS | - | - | - | - | - | - | 6 | 17 | >80 | No |
| Hazzan 2003^73^ | Israel | 67 | RCS | 29 | 15 | - | - | 17 | 6 | - | - | >80 | No |
| Tambyraja 2004^74^ | UK | 117 | RCS | - | 28 | 62 | 14 | 12 | 8 | 39 | 78 | >80 | No |
| Tambyraja 2005^75^ | UK | 76 | RCS | - | 16 | 43 | 1 | 7 | 3 | 18 | 58 | >80 | No |
| Leandros 2007^76^ | Greece | 92 | RCS | 41 | 29 | - | - | 14 | 8 | 29 | 63 | >80 | No |
| Marcari 2012^77^ | Brazil | 42 | RCS | 4 | - | 23 | - | 15 | - | 0 | 42 | >80 | No |
| Lupinacci 2013^78^ | Brazil | 81 | RCS | - | 30 | 22 | - | - | 29 | 30 | 51 | >80 | No |
| Wiggins 2018^79^ | UK | 3539 | RCS | - | 3539 | - | - | - | - | - | - | >80 | No |
| Dubecz 2012^80^ | Germany | 22 | RCS | - | 1331 | - | - | - | 1678 | 20 | 2 | >90 | No |
| Irojah 2017^81^ | USA | 1007 | RCS | - | - | - | - | - | - | 200 | 807 | >90 | No |
| Mayol 1997^82^ | Spain | 158 | RCS | 116 | 32 | - | - | 10 | - | - | - | >60 | Yes |
| Yetim 2010^83^ | Turkey | 146 | RCS | - | 22 | - | 121 | - | 3 | - | - | >60 | Yes |
| Nazeer 2012^84^ | Pakistan | 200 | RCS | - | - | - | 200 | - | - | - | - | >60 | Yes |
| Bhandari 2017^85^ | Nepal | 242 | RCS | 126 | 15 | - | 6 | 8 | 87 | - | - | >60 | Yes |
| Ekici 2018^86^ | Turkey | 665 | RCS | - | 579 | - | - | - | 86 | - | - | >60 | Yes |
| Rey 1995^87^ | Japan | 364 | RCS | - | - | - | - | - | - | - | - | >65 | Yes |
| Firilas 1996^88^ | USA | 194 | RCS | 11 | 15 | - | - | 14 | 48 | - | - | >65 | Yes |
| Lo 1996^89^ | Hong Kong | 70 | RCS | - | 70 | - | - | - | - | 70 | 0 | >65 | Yes |
| Tagle 1997^90^ | USA | 99 | RCS | 55 | 22 | - | 3 | 10 | - | - | - | >65 | Yes |
| Brunt 2001^91^ | USA | 421 | RCS | 312 | 63 | - | - | 39 | 1 | 31 | 390 | >65 | Yes |
| Bingener 2003^10^ | USA | 5884 | RCS | 173 | 73 | - | - | - | 54 | - | - | >65 | Yes |
| Majeski 2004^92^ | USA | 239 | RCS | - | - | - | - | - | - | 22 | 217 | >65 | Yes |
| Annamaneni 2005^93^ | USA | 46 | RCS | - | 15 | - | - | 4 | - | 30 | 16 | >65 | Yes |
| Kauvar 2005^94^ | USA | 315 | RCS | - | 58 | 27 | - | - | 230 | 58 | 257 | >65 | Yes |
| do Amaral 2006^95^ | Brazil | 190 | RCS | - | 190 | - | - | - | - | - | - | >65 | Yes |
| Kwon 2006^96^ | Japan | 516 | RCS | - | 66 | - | - | - | 605 | 14 | 502 | >65 | Yes |
| Kaya 2008^97^ | USA | 1992 | RCS | - | - | - | - | - | 1916 | - | - | >65 | Yes |
| Osman 2008^98^ | Turkey | 286 | RCS | - | - | - | 286 | - | - | - | - | >65 | Yes |
| Chang 2009^99^ | Taiwan | 627 | RCS | - | 84 | - | 544 | - | - | - | - | >65 | Yes |
| Kim 2009^100^ | South Korea | 388 | RCS | - | - | - | - | - | - | - | - | >65 | Yes |
| Cui 2010^101^ | China | 4048 | RCS | - | - | - | - | - | - | - | - | >65 | Yes |
| Yetim 2010^102^ | Turkey | 511 | RCS | - | 51 | - | 455 | - | 1 | - | - | >65 | Yes |
| Tucker 2011^103^ | USA | 23852 | RCS | - | 19082 | - | 4770 | - | - | 2159 | 21693 | >65 | Yes |
| Qasaimeh 2012^104^ | Jordan | 1539 | RCS | 1266 | 158 | - | - | 36 | 79 | 158 | 1381 | >65 | Yes |
| Agrusa 2014^105^ | Italy | 1227 | RCS | NR | NR | - | - | NR | NR | 230 | 997 | >65 | Yes |
| Nielsen 2014^106^ | Denmark | 14, 417 | RCS | - | - | - | 69 | - | 665 | - | - | >65 | Yes |
| Teixeira 2014^107^ | Portugal | 249 | RCS | - | 249 | - | - | - | - | - | - | >65 | Yes |
| Zhao 2015^108^ | China | 112 | RCS | - | - | - | 98 | - | 28 | - | - | >65 | Yes |
| Rizzuto 2016^109^ | Germany | 355 | RCS | - | - | - | 355 | - | - | 12 | 343 | >65 | Yes |
| Ido 1995^110^ | Japan | 712 | RCS | - | - | - | - | - | - | - | - | >70 | Yes |
| Paganini 2002^111^ | Italy | 284 | RCS | 266 | 3 | - | - | 93 | 269 | 0 | 284 | >70 | Yes |
| Yetkin 2009^12^ | Turkey | 595 | RCS | - | - | - | - | - | - | - | - | >70 | Yes |
| Fujikawa 2012^112^ | Japan | 111 | RCS | - | 111 | - | - | - | - | - | - | >70 | Yes |
| Ambe 2015^113^ | Germany | 152 | RCS | - | 152 | - | - | - | - | - | - | >70 | Yes |
| Pessaux 2000^114^ | France | 863 | PCS | - | - | - | 863 | - | - | - | - | >75 | Yes |
| Kirshtein 2008^115^ | Israel | 1216 | RCS | - | 225 | 58 | - | - | - | - | - | >75 | Yes |
| Polychronidis 2008^116^ | Greece | 2412 | RCS | - | - | - | - | - | - | 315 | 2097 | >75 | Yes |
| Su 2009^117^ | Taiwan | 56 | RCS | - | 56 | - | - | - | - | - | - | >75 | Yes |
| Lill 2011^118^ | Finland | 240 | RCS | 81 | - | - | - | - | 159 | 0 | 240 | >75 | Yes |
| Fuks 2015^119^ | France | 414 | RCS | - | 414 | - | - | - | - | - | - | >75 | Yes |
| Maxwell 1998^120^ | USA | 315 | RCS | - | - | - | - | - | - | 22 | 293 | >80 | Yes |
| Costi 2007^121^ | Italy | 160 | RCS | - | - | - | - | - | 160 | - | - | >80 | Yes |
| Pavlidis 2008^122^ | Greece | 1263 | RCS | 13 | 2 | - | - | 4 | 2 | - | - | >80 | Yes |
| Leardi 2009^123^ | Italy | 341 | RCS | - | 138 | - | - | - | 203 | - | - | >80 | Yes |
| Lee 2013^124^ | Korea | 380 | RCS | - | 73 | 307 | 31 | 19 | 18 | - | - | >80 | Yes |
| Mauro 2014^125^ | UK | 131 | RCS | - | - | - | 131 | - | - | 0 | 131 | >80 | Yes |
| Peker 2014^126^ | Turkey | 296 | RCS | - | 24 | - | 254 | 18 | - | 11 | 285 | >80 | Yes |
| Lee 2015^127^ | Korea | 302 | RCS | - | 302 | - | - | - | - | - | - | >80 | Yes |
| Palsson 2016^128^ | Sweden | 1961 | RCS | 630 | 1248 | - | - | 263 | 703 | - | - | >80 | Yes |
| Wakasugi 2017^129^ | Japan | 810 | RCS | - | - | - | - | - | - | - | - | >80 | Yes |
| Novello 2018^130^ | Italy | 355 | RCS | - | 55 | - | 106 | - | 233 | 191 | 164 | >80 | Yes |
| Yokota 2018^131^ | Japan | 351 | RCS | - | 351 | - | - | - | - | - | - | >80 | Yes |
| Priego 2014^132^ | USA | 2035 | RCS | - | - | - | - | - | - | 0 | 2035 | >85 | Yes |

*Abbreviations: PCS - Prospective cohort study, RCS - Retrospective cohort study, UK - United Kingdom, USA - United States of America*

Supplementary Table 3 Summary of Quality assessment of included studies

| **Study Name** | **Region** | **Baseline Confounding** | **Selection of participants** | **Classification of interventions** | **Deviation from intended interventions** | **Missing data** | **Measurement of outcomes** | **Selection of reported results** | **Overall risk of bias** |
| --- | --- | --- | --- | --- | --- | --- | --- | --- | --- |
| Schreiber 1978^39^ | USA | **Low** | **Moderate** | **Low** | **Low** | **Moderate** | **Low** | **Low** | **Low** |
| Loureiro 2011^40^ | Brazil | **Low** | **Low** | **Low** | **Low** | **Low** | **Low** | **Low** | **Low** |
| Serban 2016^36^ | Romania | **Low** | **Low** | **Low** | **Low** | **Low** | **Low** | **Low** | **Low** |
| Houghton 1985^41^ | UK | **Severe** | **Low** | **Low** | **Low** | **Low** | **Low** | **Moderate** | **Moderate** |
| Amato 2010^42^ | Italy | **Moderate** | **Low** | **Low** | **Low** | **Low** | **Low** | **Low** | **Low** |
| Pigott 1988^43^ | USA | **Moderate** | **Low** | **Low** | **Low** | **Low** | **Moderate** | **Low** | **Low** |
| Saxe 1993^44^ | USA | **Moderate** | **Moderate** | **Low** | **Low** | **Low** | **Low** | **Low** | **Low** |
| Askew 1995^45^ | Australia | **Severe** | **Low** | **Low** | **Low** | **Low** | **Low** | **Low** | **Moderate** |
| Escarce 1995^46^ | USA | **Low** | **Moderate** | **Low** | **Low** | **Low** | **Moderate** | **Low** | **Moderate** |
| Lujan 1998^47^ | Spain | **Low** | **Low** | **Low** | **Low** | **Low** | **Low** | **Low** | **Low** |
| Wang 2006^48^ | Taiwan | **Low** | **Low** | **Low** | **Low** | **Low** | **Low** | **Low** | **Low** |
| Malik 2007^49^ | Pakistan | **Low** | **Low** | **Low** | **Low** | **Low** | **Low** | **Low** | **Low** |
| Cheng 2008^50^ | Taiwan | **Low** | **Moderate** | **Low** | **Low** | **Moderate** | **Low** | **Moderate** | **Moderate** |
| Lasithiotakis 2012^51^ | Greece | **Low** | **Moderate** | **Low** | **Low** | **Low** | **Low** | **Low** | **Low** |
| Cull 2013^52^ | USA | **Low** | **Low** | **Low** | **Low** | **Low** | **Low** | **Low** | **Low** |
| Ferrarese 2013^53^ | Italy | **Moderate** | **Moderate** | **Low** | **Low** | **Low** | **Low** | **Low** | **Moderate** |
| Rao 2013^54^ | USA | **Low** | **Low** | **Low** | **Low** | **Low** | **Low** | **Low** | **Low** |
| Rubert 2016^55^ | Brazil | **Moderate** | **Moderate** | **Low** | **Low** | **Low** | **Low** | **Low** | **Low** |
| Zeren 2017^56^ | Turkey | **Low** | **Moderate** | **Low** | **Low** | **Low** | **Low** | **Low** | **Low** |
| Schlottmann 2018^29^ | USA | **Low** | **Moderate** | **Low** | **Low** | **Low** | **Moderate** | **Low** | **Low** |
| Huber 1983^57^ | USA | **Severe** | **Moderate** | **Low** | **Low** | **Low** | **Low** | **Low** | **Moderate** |
| Margiotta 1988^58^ | USA | **Severe** | **Low** | **Low** | **Low** | **Low** | **Low** | **Low** | **Moderate** |
| Watemberg 1997^59^ | Israel | **Severe** | **Moderate** | **Low** | **Low** | **Low** | **Low** | **Low** | **Moderate** |
| Montori 2000^60^ | Italy | **Severe** | **Low** | **Low** | **Low** | **Low** | **Moderate** | **Low** | **Moderate** |
| Fisichella 2002^61^ | Italy | **Moderate** | **Low** | **Low** | **Low** | **Low** | **Low** | **Low** | **Moderate** |
| Zappulla 2009^62^ | Italy | **Low** | **Low** | **Low** | **Low** | **Low** | **Low** | **Low** | **Low** |
| Caglia 2012^63^ | Italy | **Low** | **Low** | **Low** | **Low** | **Low** | **Low** | **Low** | **Low** |
| Aprea 2016^64^ | Italy | **Low** | **Severe** | **Low** | **Low** | **Low** | **Low** | **Low** | **Moderate** |
| Mastalerz 2018^65^ | Poland | **Low** | **Moderate** | **Low** | **Low** | **Moderate** | **Low** | **Low** | **Low** |
| Decker 2001^66^ | Belgium | **Moderate** | **Low** | **Low** | **Low** | **Low** | **Low** | **Low** | **Low** |
| Pessaux 2001^67^ | France | **Low** | **Low** | **Low** | **Low** | **Low** | **Low** | **Severe** | **Moderate** |
| Chau 2002^68^ | Hong Kong | **Low** | **Low** | **Low** | **Low** | **Low** | **Low** | **Low** | **Low** |
| Coenye 2005^69^ | Belgium | **Moderate** | **Low** | **Low** | **Moderate** | **Low** | **Low** | **Low** | **Low** |
| Moyson 2008^70^ | Belgium | **Moderate** | **Low** | **Low** | **Low** | **Low** | **Low** | **Low** | **Low** |
| Maxwell 1998^71^ | USA | **Severe** | **Low** | **Low** | **Low** | **Low** | **Moderate** | **Low** | **Moderate** |
| Uecker 2001^72^ | USA | **Low** | **Low** | **Low** | **Low** | **Low** | **Low** | **Low** | **Low** |
| Arthur 2003^9^ | UK | **Severe** | **Low** | **Low** | **Low** | **Low** | **Low** | **Low** | **Moderate** |
| Hazzan 2003^73^ | Israel | **Moderate** | **Low** | **Low** | **Low** | **Low** | **Low** | **Low** | **Low** |
| Tambyraja 2004^74^ | UK | **Moderate** | **Low** | **Low** | **Low** | **Low** | **Low** | **Low** | **Low** |
| Tambyraja 2005^75^ | UK | **Moderate** | **Low** | **Low** | **Low** | **Low** | **Low** | **Low** | **Low** |
| Leandros 2007^76^ | Greece | **Low** | **Low** | **Low** | **Low** | **Low** | **Low** | **Low** | **Low** |
| Marcari 2012^77^ | Brazil | **Moderate** | **Low** | **Low** | **Low** | **Low** | **Low** | **Low** | **Low** |
| Lupinacci 2013^78^ | Brazil | **Low** | **Low** | **Low** | **Low** | **Low** | **Low** | **Low** | **Low** |
| Wiggins 2018^79^ | UK | **Severe** | **Moderate** | **Low** | **Low** | **Low** | **Low** | **Low** | **Moderate** |
| Dubecz 2012^80^ | Germany | **Low** | **Low** | **Low** | **Low** | **Severe** | **Low** | **Low** | **Moderate** |
| Irojah 2017^81^ | USA | **Moderate** | **Low** | **Low** | **Low** | **Low** | **Moderate** | **Low** | **Low** |
| Mayol 1997^82^ | Spain | **Moderate** | **Moderate** | **Low** | **Low** | **Low** | **Low** | **Low** | **Low** |
| Yetim 2010^83^ | Turkey | **Low** | **Low** | **Low** | **Low** | **Low** | **Low** | **Low** | **Low** |
| Nazeer 2012^84^ | Pakistan | **Moderate** | **Severe** | **Low** | **Low** | **Low** | **Low** | **Low** | **Moderate** |
| Bhandari 2017^85^ | Nepal | **Low** | **Low** | **Low** | **Low** | **Low** | **Low** | **Low** | **Low** |
| Ekici 2018^86^ | Turkey | **Low** | **Low** | **Low** | **Low** | **Low** | **Low** | **Low** | **Low** |
| Rey 1995^87^ | Japan | **Moderate** | **Low** | **Low** | **Low** | **Low** | **Low** | **Low** | **Low** |
| Firilas 1996^88^ | USA | **Moderate** | **Low** | **Low** | **Low** | **Low** | **Low** | **Low** | **Low** |
| Lo 1996^89^ | Hong Kong | **Low** | **Moderate** | **Low** | **Low** | **Low** | **Low** | **Low** | **Low** |
| Tagle 1997^90^ | USA | **Moderate** | **Low** | **Low** | **Low** | **Low** | **Low** | **Low** | **Low** |
| Brunt 2001^91^ | USA | **Moderate** | **Low** | **Low** | **Low** | **Low** | **Low** | **Low** | **Low** |
| Bingener 2003^10^ | USA | **Moderate** | **Low** | **Low** | **Low** | **Low** | **Low** | **Low** | **Low** |
| Majeski 2004^92^ | USA | **Moderate** | **Low** | **Low** | **Low** | **Low** | **Low** | **Low** | **Low** |
| Annamaneni 2005^93^ | USA | **Low** | **Low** | **Low** | **Low** | **Low** | **Low** | **Low** | **Low** |
| Kauvar 2005^94^ | USA | **Moderate** | **Low** | **Low** | **Low** | **Low** | **Low** | **Low** | **Low** |
| do Amaral 2006^95^ | Brazil | **Low** | **Moderate** | **Low** | **Low** | **Low** | **Low** | **Low** | **Low** |
| Kwon 2006^96^ | Japan | **Low** | **Low** | **Low** | **Low** | **Low** | **Low** | **Severe** | **Moderate** |
| Kaya 2008^97^ | USA | **Severe** | **Low** | **Low** | **Low** | **Low** | **Low** | **Low** | **Moderate** |
| Osman 2008^98^ | Turkey | **Low** | **Moderate** | **Low** | **Low** | **Low** | **Low** | **Low** | **Low** |
| Chang 2009^99^ | Taiwan | **Low** | **Low** | **Moderate** | **Moderate** | **Moderate** | **Moderate** | **Moderate** | **Moderate** |
| Kim 2009^100^ | South Korea | **Low** | **Low** | **Low** | **Low** | **Low** | **Low** | **Low** | **Low** |
| Cui 2010^101^ | China | **Low** | **Moderate** | **Low** | **Low** | **Low** | **Low** | **Low** | **Low** |
| Yetim 2010^102^ | Turkey | **Low** | **Low** | **Low** | **Low** | **Low** | **Low** | **Low** | **Low** |
| Tucker 2011^103^ | USA | **Low** | **Moderate** | **Low** | **Low** | **Low** | **Low** | **Low** | **Low** |
| Qasaimeh 2012^104^ | Jordan | **Low** | **Moderate** | **Low** | **Low** | **Low** | **Low** | **Low** | **Low** |
| Agrusa 2014^105^ | Italy | **Low** | **Moderate** | **Low** | **Low** | **Low** | **Low** | **Low** | **Low** |
| Nielsen 2014^106^ | Denmark | **Low** | **Low** | **Low** | **Low** | **Low** | **Low** | **Low** | **Low** |
| Teixeira 2014^107^ | Portugal | **Moderate** | **Low** | **Low** | **Low** | **Low** | **Low** | **Low** | **Low** |
| Zhao 2015^108^ | China | **Low** | **Severe** | **Low** | **Low** | **Low** | **Low** | **Low** | **Moderate** |
| Rizzuto 2016^109^ | Germany | **Moderate** | **Moderate** | **Low** | **Low** | **Low** | **Low** | **Low** | **Low** |
| Ido 1995^110^ | Japan | **Low** | **Low** | **Low** | **Low** | **Low** | **Low** | **Moderate** | **Low** |
| Paganini 2002^111^ | Italy | **Low** | **Low** | **Low** | **Low** | **Low** | **Low** | **Low** | **Low** |
| Yetkin 2009^12^ | Turkey | **Moderate** | **Low** | **Low** | **Low** | **Low** | **Low** | **Low** | **Low** |
| Fujikawa 2012^112^ | Japan | **Low** | **Moderate** | **Low** | **Low** | **Low** | **Low** | **Low** | **Low** |
| Ambe 2015^113^ | Germany | **Low** | **Low** | **Low** | **Low** | **Low** | **Low** | **Low** | **Low** |
| Pessaux 2000^114^ | France | **Moderate** | **Low** | **Low** | **Low** | **Low** | **Low** | **Low** | **Low** |
| Kirshtein 2008^115^ | Israel | **Low** | **Low** | **Low** | **Low** | **Low** | **Low** | **Low** | **Low** |
| Polychronidis 2008^116^ | Greece | **Low** | **Low** | **Low** | **Low** | **Low** | **Low** | **Low** | **Low** |
| Su 2009^117^ | Taiwan | **Low** | **Low** | **Low** | **Low** | **Low** | **Low** | **Low** | **Low** |
| Lill 2011^118^ | Finland | **Low** | **Low** | **Low** | **Low** | **Low** | **Low** | **Low** | **Low** |
| Fuks 2015^119^ | France | **Low** | **Low** | **Low** | **Low** | **Low** | **Low** | **Low** | **Low** |
| Maxwell 1998^120^ | USA | **Moderate** | **Low** | **Low** | **Low** | **Low** | **Low** | **Low** | **Low** |
| Costi 2007^121^ | Italy | **Moderate** | **Moderate** | **Low** | **Low** | **Low** | **Low** | **Moderate** | **Moderate** |
| Pavlidis 2008^122^ | Greece | **Low** | **Low** | **Low** | **Low** | **Low** | **Low** | **Low** | **Low** |
| Leardi 2009^123^ | Italy | **Moderate** | **Low** | **Low** | **Low** | **Low** | **Low** | **Low** | **Low** |
| Lee 2013^124^ | Korea | **Low** | **Low** | **Low** | **Low** | **Low** | **Low** | **Low** | **Low** |
| Mauro 2014^125^ | UK | **Low** | **Moderate** | **Low** | **Low** | **Low** | **Low** | **Low** | **Low** |
| Peker 2014^126^ | Turkey | **Low** | **Low** | **Low** | **Low** | **Low** | **Low** | **Low** | **Low** |
| Lee 2015^127^ | Korea | **Low** | **Moderate** | **Low** | **Low** | **Low** | **Low** | **Low** | **Low** |
| Palsson 2016^128^ | Sweden | **Low** | **Low** | **Low** | **Low** | **Low** | **Low** | **Low** | **Low** |
| Wakasugi 2017^129^ | Japan | **Low** | **Moderate** | **Low** | **Low** | **Low** | **Low** | **Moderate** | **Low** |
| Novello 2018^130^ | Italy | **Low** | **Moderate** | **Low** | **Low** | **Low** | **Low** | **Low** | **Low** |
| Yokota 2018^131^ | Japan | **Low** | **Moderate** | **Low** | **Low** | **Low** | **Low** | **Low** | **Low** |
| Priego 2014^132^ | USA | **Moderate** | **Moderate** | **Low** | **Low** | **Low** | **Low** | **Low** | **Low** |

*Abbreviations: UK - United Kingdom, USA - United States of America*

**Supplementary Figure 1 Impact of age on overall complications in patients**

**undergoing laparoscopic cholecystectomy**

**Supplementary Figure 2 Impact of age on major complications in patients**

**undergoing laparoscopic cholecystectomy**

**Supplementary Figure 3 Impact of age cut-offs on major complications in patients undergoing laparoscopic cholecystectomy (A) ≥65 years (B) ≥70 years (C) ≥75 years (D) ≥80 years**

**Supplementary Figure 4 Impact of age on conversion to open in patients**

**undergoing laparoscopic cholecystectomy**

**Supplementary Figure 5 Impact of age cut-offs on conversion to open in patients undergoing laparoscopic cholecystectomy (A) ≥60 years (B) ≥65 years (C) ≥70 years (D) ≥75 years (E) ≥80 years**

**Supplementary Figure 6 Impact of age on bile leaks in patients undergoing**

**laparoscopic cholecystectomy**

**Supplementary Figure 7 Impact of age cut-offs on bile leaks in patients undergoing laparoscopic cholecystectomy (A) ≥60 years (B) ≥65 years (C) ≥70 years (D) ≥75 years (E) ≥80 years**

**Supplementary Figure 8 Impact of age on postoperative mortality in patients**

**undergoing laparoscopic cholecystectomy**

**Supplementary Figure 9 Impact of age on length of stay in patients undergoing**

**laparoscopic cholecystectomy**

**Supplementary Figure 10 Impact of age cut-offs on length of stay in patients**

**undergoing laparoscopic cholecystectomy (A) ≥65 years (B) ≥70 years (C) ≥75**

**years (D) ≥80 years**

**Supplementary Figure 11 Funnel plot on the impact of age on major**

**complications in patients undergoing laparoscopic cholecystectomy**

**Supplementary Figure 12 Funnel plot on the impact of age on conversion to**

**open in patients undergoing laparoscopic cholecystectomy**

**Supplementary Figure 13 Funnel plot on the impact of age on bile leaks in**

**patients undergoing laparoscopic cholecystectomy**

**Supplementary Figure 14 Funnel plot on the impact of age on postoperative**

**mortality in patients undergoing laparoscopic cholecystectomy**

**Supplementary Figure 15 Funnel plot on the impact of age on length of stay in**

**patients undergoing laparoscopic cholecystectomy**

**Supplementary Figure 16 Funnel plot on the impact of age on overall**

**complications in patients undergoing laparoscopic cholecystectomy**
